# Supplementary material for: Theory-based electronic learning intervention to support appropriate antibiotic prescribing by nurse and pharmacist independent prescribers: an acceptability and feasibility experimental study using mixed methods
Source: BMJ Open. 2020 Jun 30;10(6):e036181. doi: 10.1136/bmjopen-2019-036181 (PMC7328741; doi:10.1136/bmjopen-2019-036181)
Supplement: Author's manuscript [file bmjopen-2019-036181.draft_revisions.pdf]

BMJ Open is committed to open peer review. As part of this commitment we make the peer review history of every article we publish publicly available.

When an article is published we post the peer reviewers' comments and the authors' responses online. We also post the versions of the paper that were used during peer review. These are the versions that the peer review comments apply to.

The versions of the paper that follow are the versions that were submitted during the peer review process. They are not the versions of record or the final published versions. They should not be cited or distributed as the published version of this manuscript.

BMJ Open is an open access journal and the full, final, typeset and author-corrected version of record of the manuscript is available on our site with no access controls, subscription charges or pay-per-view fees (http://bmjopen.bmj.com).

If you have any questions on BMJ Open's open peer review process please email info.bmjopen@bmj.com

BMJ Open

A theory-based electronic learning intervention to support appropriate antibiotic prescribing by nurse and pharmacist independent prescribers: an acceptability and feasibility study.

Journal:	*BMJ Open*
Manuscript ID	bmjopen-2019-036181
Article Type:	Original research
Date Submitted by the Author:	03-Dec-2019

Complete List of Authors:	Lim, Rosemary; Reading University, School of Pharmacy<br>Courtenay, Molly; Cardiff University, Healthcare Sciences<br>Deslandes, Rhian<br>Ferriday, Rebecca; Cardiff University<br>Gillespie, David; Centre for Trials Research, University of Cardiff ,<br>Hodson, Karen; Cardiff University<br>Reid, Nicholas; Public Health Wales,<br>Thomas, Neil; Cardiff University<br>Chater, Angel; University of Bedfordshire, Department of Sport Science and Physical

Keywords:	EDUCATION & TRAINING (see Medical Education & Training), PRIMARY CARE, Respiratory infections < THORACIC MEDICINE

SCHOLARONE™
Manuscripts

A theory-based electronic learning intervention to support appropriate antibiotic prescribing by nurse and pharmacist independent prescribers: an acceptability and feasibility study.

Rosemary Lim, Associate Professor in Medication Use and Safety, Reading School of Pharmacy, University of Reading, PO Box 226, Whiteknights, Reading, Berkshire RG6 6AP, UK

Molly Courtenay, Professor of Health Sciences, School of Healthcare Sciences, Cardiff University, Cardiff, CF24 OAB, UK

Rhian Deslandes, Senior Lecturer, Cardiff School of Pharmacy and Pharmaceutical Sciences, Cardiff University, Cardiff, CF10 3NB, UK

Rebecca Ferriday, Learning Technology Manager, School of Healthcare Sciences,

Cardiff University, Cardiff, CF24 OAB, UK

David Gillespie, Deputy Director of Infection, Inflammation & Immunity Trials, Senior Research Fellow, Centre for Trials Research

Cardiff University, Cardiff, CF24 OAB, UK

Karen Hodson, Senior Lecturer, Cardiff School of Pharmacy and Pharmaceutical Sciences, Cardiff University, Cardiff, CF10 3NB, UK

Nicholas Reid, All Wales Consultant Antimicrobial Pharmacist, Healthcare Associated Infection, Antimicrobial Resistance & Prescribing Programme (HARP team),

Public Health Wales, Cardiff, CF10 4BZ, UK

Neil Thomas, Lecturer, School of Healthcare Sciences,

Cardiff University, Cardiff, CF24 OAB, UK

Angel Chater, Reader in Health Psychology and Behaviour Change

University of Bedfordshire, Bedford, MK419EA, UK, University College London School of Pharmacy, London, WC1H 9JP, UK

Corresponding author

Rosemary Lim, Email: r.h.m.lim@reading.ac.uk

For peer review only

Abstract

Objectives: To assess the acceptability and feasibility of using a theory-based electronic learning intervention designed to support appropriate antibiotic prescribing by nurse and pharmacist independent prescribers for patients presenting with common, acute, uncomplicated self-limiting respiratory tract infections (RTIs).

Design: Experimental with mixed methods; pre- and post-intervention online surveys and semi-structured interviews.

Setting: Primary care settings across the United Kingdom.

Participants: 11 nurse and 4 pharmacist prescribers.

Intervention: A theory-based brief interactive animation electronic learning activity comprised of a consultation scenario by a prescriber with an adult presenting with a common, acute, uncomplicated self-limiting RTI.

Outcome measures: Recruitment, response and attrition rates were assessed. Overall usefulness of the intervention was assessed by analysing prescribers' self-reported confidence and knowledge in treating patients with RTIs before and after undertaking the intervention, and views on the relevance of the intervention to their work. Acceptability of the intervention was assessed in semi-structured interviews. The feasibility of data collection methods was assessed by recording the number of study components completed by prescribers.

Results: 15 prescribers (maximum sample size) consented and completed all four stages of the study. Prescribers reported high to very high levels of confidence and knowledge pre- and post-intervention, with slight post-intervention increases in communicating with patients and a slight reduction in building rapport. Qualitative findings supported quantitative findings; prescribers were reassured of their own practice which in turn increased their confidence and knowledge in consultations. The information in the intervention was not new to prescribers but was applicable and useful to consolidate learning and enable self-reflection. Completing the e-learning intervention was acceptable to prescribers.

For peer review only

Conclusions: It was feasible to conduct the study. The intervention was acceptable and useful to prescribers. Future work will review the clinical content in the intervention before conducting a full trial.

Strengths and limitations of this study

- To our knowledge, this study was the first to examine the acceptability and feasibility of using an electronic, interactive, animation-based learning intervention to support appropriate antibiotic prescribing by nurse and pharmacist independent prescribers for patients presenting with common, acute, uncomplicated self-limiting respiratory tract infections.
- A mixed-methods approach allowed for validation of quantitative findings; interview findings enabled a richer picture to the contextual factors affecting the feasibility and acceptability of the intervention.
- The successful recruitment of nurse and pharmacist prescribers and completion of the intervention demonstrated the acceptability and feasibility of using the intervention amongst nurse prescribers.

Keywords: antibiotics, prescribing, nurse prescribers, pharmacist prescribers, electronic learning, antimicrobial stewardship

For peer review only

Introduction

Each year, antimicrobial resistant (AMR) infections cause approximately 700,000 deaths globally. By 2050 it has been predicted that this will rise to 10 million, combined with a cumulative cost of $100 trillion [1]. In the European Union and the European Economic Area, the figure is an estimated 33 110 deaths, 875 000 disability-adjusted life-years [2] and €1.5 billion in extra healthcare costs [3]. The inappropriate use of antimicrobials in humans is one of the leading drivers for the growth of AMR [4] and strategies to support appropriate antibiotic use are important. A key global strategy is careful stewardship of antibiotics [1]. Antimicrobial stewardship (AMS), comprises "a collection of co-ordinated interprofessional focused strategies to optimise antibiotic use by ensuring that every patient receives an antibiotic only when it is clinically indicated and then receives the appropriate antibiotic, at the right dose, duration and route of administration" [5].

For peer review only

Unless there is serious underlying comorbidity, common, acute, uncomplicated self-limiting respiratory tract infections (RTIs) usually resolve spontaneously, with antibiotics in most cases, unlikely to offer clinical benefit [6]. Despite this, more than 60% of all prescriptions issued in the United Kingdom (UK) primary care are for RTIs [7, 8]. As well as contributing to the spread of resistance, their unnecessary use also puts patients at risk of side effects [9]. A global priority that has been recognised for some time is the need to conserve antibiotic sensitivity by managing RTIs without recourse to antibiotics and healthcare professionals who routinely prescribe antibiotics is a key target for interventions [9-12].

Multifaceted interventions that address barriers to change in specific healthcare settings involving active education strategies, feedback on antibiotic prescribing behaviour and seeking to improve prescribing for all as opposed to specific respiratory infections, tend towards greater effectiveness in medical prescribers [13-14]. Comparable reductions in the utilisation of antibiotics by medical prescribers have been demonstrated using these strategies via electronic learning [15].

Appropriately qualified nurses and pharmacists in the UK can prescribe medicines independently and around 34,000 nurses and 8,000 pharmacists have independent prescribing capability [16]. Nurse and pharmacist prescribers frequently manage patients with RTIs and prescribe around 8% of all primary care antibiotics dispensed in England [17]. As compared with medical prescribers, a broader range of factors influence the prescribing behaviour of these professionals [18]. Factors include diagnostic uncertainty and the patient's clinical condition [18-21], the expectations of patients for an antibiotic [18-19, 21], relationships with other prescribers and knowledge of current guidelines [18-19, 22]. Interventions are available to support the various AMS activities that healthcare professionals are involved in [23-24] however, no interventions exist to specifically support appropriate antibiotic prescribing behaviour by nurse and pharmacist independent prescribers. We developed an intervention to support appropriate antibiotic prescribing by nurse and pharmacist independent prescribers. The aim of this paper is to describe the findings of a study designed to assess the acceptability and feasibility of a theory-based electronic learning intervention designed to support appropriate antibiotic prescribing by nurse and pharmacist independent prescribers when treating patients presenting with RTIs.

Method

Ethical considerations

The School of Healthcare Sciences Research Governance and Ethics Committee, Cardiff University gave ethical approval for the conduct of this study (reference: 427REC). All participants gave written informed consent before participating in the study.

Design

A mixed-methods study design was used: participants completed an online pre- and post-intervention survey, participated in the intervention and a semi-structured interview with a researcher.

For peer review only

Participant recruitment

Potential participants were UK primary care nurse and pharmacist non-medical independent prescribers (hereafter described as prescribers) who managed patients with RTIs.

Recruitment took place during November to December 2018 via several routes until a maximum of 15 nurse and pharmacist prescribers in total had been recruited; a sample size expected to enable qualitative data saturation [16]: 1. MC emailed prescribers (4 pharmacists and 17 nurses) who consented to be contacted after taking part in previous research led by MC [18], 2. KH and RL approached key contacts within their existing prescriber networks who sent out information about the study via email (approximately 195 prescribers and nurses combined). MC or RL emailed the participant information sheet and consent form to interested prescribers and encouraged them to ask any questions they may have about the study prior to making an informed choice about participating in the study. Participants gave written consent prior to taking part in the study.

Intervention

The theory-based intervention comprised a five-minute, interactive, animated scenario of a consultation by a prescriber with an adult presenting with a common, acute, uncomplicated self-limiting RTI. The development of the intervention is published elsewhere [16] and draws from previous work with nurse prescribers [25, 26] and pharmacists [27, 28]. Prescribers were sent a weblink to the intervention that was accessible on any internet-enabled device.

The intervention started with a consultation by a prescriber to reach a no antibiotic prescribing decision whereby patient-centred approaches [29] such as holistic care, individualised care, respectful care and empowering care, to reach a prescribing decision were absent. The overall patient experience and satisfaction was poor; the patient was not confident in the treatment decision made in the consultation. This is followed by a second scenario where a prescriber used a patient-centred motivational interviewing style [30] to reach a no antibiotic prescribing decision in the consultation. In this scenario, the patient left the consultation satisfied and confident with the treatment received. To facilitate active

For peer review only

learning, a range of open and closed questions were also incorporated within the e-learning activity.

Measures and data collection

The study was conducted in four stages in the following order: (1) pre-intervention online questionnaire, (2) e-learning intervention, (3) post-intervention online questionnaire and (4) semi-structured telephone interviews. Overall usefulness of the intervention was assessed by analysing prescribers' self-reported confidence and knowledge in treating patients with RTIs before and after undertaking the intervention, and views on the relevance of the intervention to their work. Acceptability of the intervention was assessed in semi-structured interviews. The feasibility of data collection methods was assessed by recording the number of study components completed by prescribers.

Pre-post intervention online questionnaires

Prescribers were sent a link and completed an online questionnaire before and immediately after completing the intervention, aimed to assess their perceptions of the impact of the intervention. The questionnaires were developed based on findings from our previous work [16]. The pre-intervention questionnaire assessed prescribers' knowledge and confidence prescribing antibiotics for patients presenting with RTIs. Prescribers were asked to rate their responses on six items using a 5-point Likert scale (Strongly Disagree to Strongly Agree): (1) gain information on patient expectations, (2) support patients, (3) build rapport, (4) communicate effectively, (5) see and examine different viewpoints, (6) ensure patients both understand and are happy with the prescribing decision. The post-intervention questionnaire contained the same questions as the pre-intervention questionnaire, and six additional questions about the usefulness of the intervention: (1) whether the information was known to participants, (2) its applicability to practice, (3) whether the intervention would be useful to them as prescribers, (4) whether it makes them feel more comfortable when speaking with patients with RTIs, (5) if it encourages participants to consider how they would apply the information to practice and (6) think differently. In both questionnaires,

demographic details were also collected: type of prescriber (i.e. nurse or pharmacist), length of time qualified as a prescriber, time in current post, clinical setting and length of consultation time.

Semi-structured interviews

Following completion of the intervention and the post-intervention questionnaire, prescribers were invited to take part in a semi-structured telephone interview to understand prescribers' experiences of using the intervention. The interview took place within 1-2 weeks post intervention to ensure retention of information. Interviews (see Box 1 for the interview schedule) explored each component of the COM-B model, the hub of the Behaviour Change Wheel [31] to create a behavioural diagnosis to understand Capability (e.g. knowledge, skills), Opportunity (e.g. social influence, environment) and Motivation (e.g. beliefs about consequences and emotional responses). Demographic details such as job title, practice setting, years qualified as a prescriber and approximate frequency of RTI consultations and antibiotic prescribing were also collected in the interview. All interviews were digitally recorded and then transcribed verbatim with identifying information removed.

Box 1 Interview schedule

**1) Capability**<br>*Examples of prompts:*<br>▪ Did the learning resource increase your knowledge and skills about prescribing antibiotics for respiratory tract infections?<br>▪ (if yes) Can you tell me in what ways?<br>▪ (if no) Can you tell me why this was the case for you?<br>▪ Did it increase your awareness of potential solutions to any difficulties you may have experienced managing the consultations of patients with RTIs?<br>▪ (if yes) Can you tell me in what ways?<br>▪ (if no) Can you tell me why this was the case for you?<br>▪ How do you think your practice will change based on the training resource?
**2) Opportunity (relating to the resource and also behaviour to be changed)**

For peer review only

Examples of prompts:<br>▪ How do you think this resource would change the norms of practice? Or the Practice of others. How will this fit in to current practice? (ie in terms of time)<br>▪ How did the resource address any gaps in your prescribing practice?<br>▪ Is the resource an acceptable/feasible delivery method that can be integrated into daily practice?
**3) Motivation**<br>*Examples of prompts:*<br>▪ In what way does the resource provide a means by which you can reflect on and develop your practice?<br>▪ How do you perceive that this resource could improve prescribing practice generally?<br>▪ How did it make you feel about their current and future practice?
**4) Other questions**<br>▪ What stood out for you the most, in the learning resource? E.g. was it the poster – was it that she came to greet the patient etc…<br>▪ How can the learning resource be improved? Was there anything that you would change for future training?

Data analysis

Pre-post intervention online questionnaires

Data were analysed using SPSS version 25 [32]. Descriptive statistics were used to characterise prescribers (frequencies and percentages), prescribers' knowledge and confidence in prescribing antibiotics for patients presenting with RTIs (mean, SD) and their views on the usefulness of the intervention (mean, SD).

Semi-structured interviews

Interviews were analysed using inductive thematic analysis [33]. Coding and categorising of data was conducted by RL using NVivo 10 [34]. Themes were then identified reviewed with a second researcher (MC) and any differences in interpretation were resolved through discussion to increase the trustworthiness of research data [35-36]. An iterative process of comparing both the quantitative and qualitative datasets allowed for data triangulation and

For peer review only

confirmed the accuracy of findings in the interviews, providing further insight to the phenomenon under study [33, 37].

Patient and public involvement

Patients and the public were not involved in the development of the research question, study design, recruitment and conduct of the study.

Results

A total of 15 of the 216 prescribers approached (7%) responded to the study invitation, consented and completed all four stages of the study between October and December 2018. The maximum number of participants targeted for the study was reached. Table 1 shows the demographic data of prescribers. Most prescribers worked in general practice (*n*=10, 67%) and had been qualified for at least two years (*n*=12, 80%). Except for prescribers in the community care setting, most prescribers worked within 10 to 30 minutes patient appointment times. Interviews with prescribers lasted between 7 and 28 minutes (mean = 16 minutes).

Table 1: Demographic data of participants (n=15)

**Type of non-medical prescriber**	
Nurse	11 (73.3%)
Pharmacist	4 (27.7%)
**Time qualified as prescriber**	
13months to 2 years	3 (20.0%) *
3-5 years	5 (33.3%)
6-10 years	2 (13.3%)
>10 years	5 (33.3%)
**Time in post**	
13 months to 2 years	4 (26.7%)
3-5 years	9 (60.0%)
6-10 years	0 (0.0%)
>10 years	2 (13.3%)
**Type of clinical setting and length of patient appointments**	
Community care (45-90 minutes)	2 (13.3%)

For peer review only

General practice (10-15 minutes)	10 (66.7%) #
Out of hours (10-20 minutes)	1 (6.7%)
General practice and out of hours (10-30 minutes)	1 (6.7%)
Urgent care (15 minutes)	1 (6.7%)

Note: * Two of the three participants were pharmacist. # All pharmacists worked in general practice and had 15 minutes appointment times.

Impact of intervention on prescribing practice

Table 2 shows prescribers' scores relating to their confidence in managing patients presenting with RTIs pre- and post-intervention. High to very high levels of confidence were reported for all statements both pre- and post-intervention. There was an increase in confidence levels in statements 1.2 (supporting patients understand health information given), 1.4 (skills to communicate with patients) and 1.5 (skills to help patients see and examine different viewpoints). Participants scored very highly for statement 1.3 (building rapport with patients) both pre- and post-intervention but there was a slight reduction in confidence post-intervention. Prescribers' confidence in their own ability to gain health-related information and that patients understand and are happy with their prescribing decision stayed the same.

Table 2: Confidence in treatment management of patients with RTI pre and post-intervention.

	Pre-intervention		Post-intervention		Mean change
**Statement**	**Min, Max**	**Mean (SD)**	**Min, Max**	**Mean (SD)**	
1.1 Confidence in gaining health-related information	3, 5	4.20 (0.68)	3, 5	4.20 (0.56)	0
1.2 Confidence in supporting patients understand health information given	4, 4	4.00 (0.00)	4, 5	4.27 (0.46)	0.27
1.3 Confidence in building rapport with patients	4, 5	4.53 (0.52)	4, 5	4.47 (0.52)	-0.06
1.4 Confidence in skills to communicate with patients	4, 5	4.53 (0.52)	3, 5	4.60 (0.63)	0.07
1.5 Confidence in skills to help patients see and examine different viewpoints	3, 5	3.87 (0.74)	3, 5	4.13 (0.64)	0.26

For peer review only

1.6 Confidence that patients understand and happy with prescribing decision	2, 5	4.07 (0.80)	3, 5	4.07 (0.46)	0

SD = standard deviation

Interviews with prescribers supported quantitative findings and revealed that watching and learning 'good practice', as shown in the intervention, reassured them of their own practice. It appeared to increase their confidence in their current practice and in refusing to prescribe antibiotics, when appropriate.

> *"So, when I watched it, I was thinking, well, yeah, I do, it reinforced what I was doing was right… and I just, you know, keep doing that." Nurse 10*

> *"It's given me more confidence in my approach really, to managing those patients with the respiratory tract infections." Pharmacist 4*

For peer review only

Usefulness of intervention

Table 3 shows prescribers' level of agreement with statements relating to the usefulness of the intervention. Responses were recorded on a 5-point Likert scale: 1= strongly disagree, 2 = somewhat disagree, 3 = neither agree nor disagree, 4 = somewhat strongly agree and 5 = strongly agree. Although prescribers disagreed that the information in the intervention was new to them, they agreed that the intervention was applicable and would be useful to them as prescribers.

Table 3: Usefulness of intervention from post-intervention questionnaire

Statement	Minimum	Maximum	Mean	Std. Deviation
2.1 The information was mostly new to me	1	2	1.33	0.49
2.2 The intervention was applicable to my practice	2	5	4.60	0.83
2.3 The intervention will be useful to me as a prescriber	3	5	4.20	0.78

For peer review only - http://bmjopen.bmj.com/site/about/guidelines.xhtml

2.4 The intervention has made me feel more comfortable speaking with patients with RTIs	2	4	3.13	0.83
2.5 The intervention has encouraged me to consider how I would apply the information in my practice	2	5	3.87	0.99
2.6 The intervention has encouraged me to think differently	1	4	3.07	1.10

Qualitative data was consistent with the questionnaire data; the majority of prescribers did not think that they had acquired new knowledge or skills from the e-learning intervention because they were largely an experienced group of practitioners. For most prescribers, however, the intervention was useful in helping them refresh their memories, consolidate learning and prompted self-reflection on prescribing for patients presenting with respiratory tract infections.

"I've been doing it quite a long time now and, you know, especially in my respiratory clinic, so I suppose, some of it was just reinforcing what I already sort of knew, but then that's good as well. It's still teaching, isn't it? It's still learning?" Nurse 6

"Yeah, I think it's about, you know, I like to do snippets of education and I sell it as… and this is very much this. It's the cement between the bricks. Your day's bricks and this is just a little filler that builds it all together." Nurse 3

"I think to… to quite a big extent, I've probably been prescribing more than I needed to…I often deal with the very frail and elderly. I often deal with the very frail and elderly. And urine infections the whole time… in the past, I have really thought, oh, crikey, they're old, they must have antibiotics. But actually, that's not necessarily a good thing." Nurse 11

Some learnt other methods of communicating with patients, for example, the use of additional information such as posters and leaflets and scheduling follow-up appointments after the consultation as a 'safety net'.

For peer review only

"I would never really have thought to... to contact the patients... I'll think about doing that in the future. And who... who are worried, um, about not prescribing antibiotics for them, um, I think that... that would ease the pressure from it." Pharmacist 4

Despite claiming not learning new knowledge, some prescribers said they intended to make changes to their practice specifically around involving the patient more in decision-making and directing patients to relevant information resources. A few prescribers claimed to have already changed their practice, for example, in considering different approaches to arrive at treatment decisions and using different ways of communicating with patients.

"I'll be sort of more vigilant in making sure that, you know, the patient understands why, I've, you know, decided to go with a certain treatment and sort of try and get the patient to engage and agree with that decision…So they're involved within the decision and I think that kind of gives them a better understanding." Pharmacist 1

"…having a simple resource that I can always apply to my consultations…was actually really helpful for that and I've used it in that context a number of times with different patients. With palpable different outcomes really, I think, you know. I have, you know, embarked on the education, rather than prescription approach, in… in consequence to doing it, so it has been good for that." Nurse 4

"I began to think much more clearly in the terms of, give me a reason to give them antibiotics. So I think that was a little bit of a theme change for me. Because I'd always thought, oh, I don't want to upset… well, I don't like upsetting people. I don't. I like people to go away feeling as if I've listened to them… And they maybe have not got what they came in to look for, but they're satisfied." Nurse 11

Acceptability of the intervention

Design and content of the intervention

Prescribers said they liked the use of e-learning because there was flexibility of where and when learning can take place. The use of 'cartoon' characters and scenes generated mixed

For peer review only

views. The scenario-based learning approach was well-received; the scenario was realistic and memorable.

> *"And that will… stay with me. You know, when I think about it, that's the vision that I have, is this person typing really fast. It's just… it was just funny. I just thought, God, that's all of us. We're just typing really fast, thinking come on! Hurry up, hurry up!" Nurse 1*

> *"I liked the scenario around it as that seemed real. And it's… it's reassuring actually those conversations that were had in the video, which we all know we all have…It kind of just said, you know, we do have these difficult things. You know, patients sometimes come in, they want antibiotics and they… you know, they perceive the need for them, but it was nice to see that we can say, you don't need them, it's okay." Nurse 3*

Prescribers said the messages presented in the e-learning intervention were appropriate, relevant, easy to understand and consistent with their own previous learning and practice. Some messages that stood out included managing patient expectations by using posters in the waiting room and providing self-care advice.

> *"I know and I know that, um, my GP colleagues also, we all struggle with the patient expectation of antibiotics, so I liked very much the idea of managing expectations of having… having the posters in the waiting room, of having the literature available." Nurse 2*

Some prescribers highlighted the use of effective communication and observational skills in the intervention to be valuable aspects of the intervention.

> *"…you know when she tells the, er, patient the second time round why she's not giving a… why she doesn't need antibiotics… I think that that part really stood out for me. Because I think it was explained clearly, it was precise, um, it wasn't too long, it*

For peer review only

wasn't too short. And it was just… just right and I think that the level of language used. What was… I really liked it. I think it was spot on really." Pharmacist 1

Completing the intervention

All the prescribers said it was quick (approximately 5 to 20 minutes) to complete the intervention. Around half of the prescribers said they completed the intervention at work for example during their lunch break, in between appointments whilst the rest completed the intervention in their own time. Despite this, prescribers agreed that the intervention could be completed during work time because it was a short learning session.

Suggestions for improving the e-learning intervention

Perceived and potential demographic of learners

Most prescribers said that the content in the e-learning intervention was pitched at a lower level of experience and suggested additional groups of people who may benefit from it such as GPs, GP trainees, new medical and NMPs, undergraduate healthcare students and the public.

"I thought at times that the level was a little bit lower. I thought the level being pitched was a little bit low and I don't know if that was intentional." Nurse 3

"I suppose what the content of the… the little video was, was fairly sort of low level, in terms of antimicrobial prescribing. I suppose it's the sort of thing that I would expect maybe most people prescribing in that environment to know." Nurse 9

Access

A few prescribers reported experiencing problems accessing the intervention at work due to an NHS firewall. They circumvented the problem by using their own personal device.

For peer review only

Overall presentation

A few prescribers suggested using a more diverse set of characters e.g. gender, race, age, and a more realistic layout of a consulting room.

Content

Other suggestions included adding learning outcomes to set learners' expectations, providing additional information such as an estimated time to complete the intervention, context to the scenario and patient history, more information about aspects shown in the learning intervention, for example, relevance and information about the poster, physical examinations, highlight key messages and including questions on self-reflection within the intervention. There was also a suggestion to include subtitles to the scene. Some prescribers suggested adding scenarios with different challenges for example, a difficult patient, different groups of patients, patient re-visits. To accommodate different learner requirements, some prescribers suggested including different levels of clinical content such as the most up-to-date clinical guidelines and additional reading materials.

Discussion

Completing the e-learning intervention was acceptable to prescribers. It was also feasible to collect study data in four separate stages. Overall, prescribers reported positive views about the usefulness of the intervention. Prescribers reported high levels of confidence in managing patients with RTIs both pre- and post-intervention. Whilst recruitment of prescribers to the study was successful, the recruitment of pharmacist prescribers was challenging. Nationally, there are fewer pharmacist prescribers compared to nurse prescribers [17] and further consideration to sample size will be needed to inform the next stage of this study.

The intervention component of the study was short, around 5 minutes, and all prescribers were able to complete the intervention at a time and place that suited them. Although some prescribers chose to complete the intervention in their personal time, all agreed that it was feasible to complete the intervention as part of their day-to-day work suggesting the

For peer review only

acceptability of using the intervention as part of their training. There was overall support for using an e-learning mode of delivery and this was consistent with the suggestion made by a similar group of prescribers [15] however, there were reported issues with accessing the intervention on NHS computers. The next iteration of the intervention will need to consider wider access issues if the learning were to take place as part of routine training in the future.

Prescribers reported high to very high levels of confidence for all statements both pre- and post-intervention and the finding was consistent with the qualitative dataset. The findings could be explained by the demographics of the study population – a group of prescribers who were largely very experienced in their role; nurse and pharmacist prescribers have often undertaken postgraduate specialist training and although many nurse prescribers have undertaken specialist qualifications, they have not all done so and only allowed to prescribe within their area of competence [38-40]. Although there were small increases in confidence levels related to skills relating to communicating with patients and a slight reduction in building rapport with patients, it was not possible to make generalisations due to the small sample size. It is however worth noting that prescribers reported that the intervention enabled self-reflection and the slight reduction in confidence in building rapport with patients could be the result of prescribers' reflection of their practice.

Regarding usefulness of the intervention, prescribers scored low on aspects relating to learning new information in the intervention. But, prescribers generally considered the intervention to be useful because it provided the opportunity to remind themselves on the topic, enabled self-reflection and change practice. The next stage of the study will review the clinical content in the intervention.

Conclusions

The study showed that it was feasible to conduct the study and the intervention was acceptable and useful to prescribers. Suggestions to improve the usefulness of the intervention focused on the clinical content rather than its delivery. Future work will consider reviewing the clinical content in the intervention before considering a full trial.

For peer review only

Acknowledgements

We would like to thank all the prescribers who took part in the study, and network contacts who helped to disseminate information about the study.

For peer review only

References

1. Her Majesty's (HM) Government (2019). Tackling antimicrobial resistance 2019-2024 – The UK's five-year national action plan. Accessed 11 February 2019
2. Cassini A, Högberg LD, Plachouras D, Quattrocchi A, Hoxha A, Simonsen GS, Colomb-Cotinat M, Kretzschmar ME, Devleesschauwer B, Cecchini M, Ouakrim DA, Oliveira TC, Struelens MJ, Suetens C, Monnet DL, and the Burden of AMR Collaborative Group. (2019) Attributable deaths and disability-adjusted life-years caused by infections with antibiotic-resistant bacteria in the EU and the European Economic Area in 2015: a population-level modelling analysis. Lancet Infect Dis 19: 56–66 http://dx.doi.org/10.1016/ S1473-3099(18)30605-4
3. European Commission - Fact Sheet. New EU action plan on antimicrobial resistance – questions and answers: Brussels 2017.
4. Holmes AH, Moore LSP, Sundsfjord A, et al.Understanding the mechanisms and drivers of antimicrobial resistance. Lancet. 2015;6736: Available at: http://linkinghub.elsevier.com/retrieve/pii/S0140673615004730
5. Manning ML, Pfeiffer J, Larson EL. Combating antibiotic resistance: The role of nursing in antibiotic stewardship. Am J Infect Control 2015; 44(12):1454-1457
6. NICE. Short clinical guidelines technical team. respiratory tract infections – antibiotic prescribing. prescribing of antibiotics for self-limiting respiratory tract infections in

adults and children in primary care. London National Institute for Health and Clinical Excellence; 2008. https://www.nice.org.uk/guidance/cg69

7. Gulliford MC, van Staa T, Dregan A, McDermott L, McCann G, Ashworth M, Charlton J, Little P, Moore MV, Yardley L (2014) Electronic health records for intervention research: a cluster randomized trial to reduce antibiotic prescribing in primary care (eCRT study). Ann Fam Med. 12(4):344-51. doi: 10.1370/afm.1659
8. Public Health England (2014) English Surveillance Programme for Antimicrobial Utilisation and Resistance *(ESPAUR) Report 2010 to 2013.*
9. Goossens H, Ferech M, Vander Stichele R, *et al.* (2005)Outpatient antibiotic use in Europe and association with resistance: a cross-national database study. Lancet, **365**:579-87.
10. Costelloe C, Metcalfe C, Lovering A, *et* al. (2010) Effect of antibiotic prescribing in primary care on antimicrobial resistance in individual patients: Systematic review and meta-analysis. BMJ, 340:c2096.
11. Sanders S, Glasziou PP, Del Mar CB, Rovers MM. Antibiotics for acute otitis media in children. Cochrane Database of Systematic Reviews 2004, Issue 1. Art. No.: CD000219. DOI: 10.1002/14651858.CD000219.pub2
12. WHO. Antimicrobial resistance Fact sheet No. 194. 2016. http://www.who.int/mediacentre/factsheets/fs194/en/ World Health Organization. Global action plan on antimicrobial resistance, 2015
13. Arnold SR, Straus SE. (2005) Interventions to improve antibiotic prescribing practices in ambulatory care. Cochrane Database Syst Rev. Issue 4. Art. No.: CD003539. DOI: 10.1002/14651858.CD003539.pub2
14. Ranji, S,Steinman, M, Shojania, K.,Gonzales, R., (2008) Interventions to Reduce Unnecessary Antibiotic Prescribing: A Systematic Review and Quantitative Analysis Medical Care, 46(8): 847-862 doi: 10.1097/MLR.0b013e318178eabd 018; bjgp18X697889. DOI: https://doi.org/10.3399/bjgp18X697889
15. Little P, Stuart B, Francis N, Douglas E, Tonkin-Crine S, Anthierens S, et al. (2013) Effects of internet-based training on antibiotic prescribing rates for acute respiratory-tract infections: a multinational, cluster, randomised, factorial, controlled trial. Lancet, 382:1175–82.

For peer review only

16. Courtenay M, Lim R, Deslandes R, et al. (2019) Theory-based electronic learning intervention to support appropriate antibiotic prescribing by nurses and pharmacists: intervention development and feasibility study protocol. BMJ Open, 9:e028326. doi:10.1136/ bmjopen-2018-028326
17. Courtenay M, Gillespie D, Lim, R. (2017) Patterns of dispensed non-medical prescriber prescriptions for antibiotics in primary care across England: a retrospective analysis. Journal of Antimicrobial Chemotherapy. dkx230, https://doi.org/10.1093/jac/dkx230
18. Courtenay M, Rowbotham S, Lim R, et al. (2019).Examining influences on antibiotic prescribing by nurse and pharmacist prescribers: a qualitative study using the Theoretical Domains Framework and COM-B. BMJ Open;9:e029177. doi:10.1136/ bmjopen-2019-029177
19. Rowbotham S, Chisholm A, Moschogianis S, Chew-Graham C, Cordingley L, Wearden A, Peters S. (2012) Challenges to nurse prescribers of a no-antibiotic prescribing strategy for managing self-limiting respiratory tract infections. J Adv Nurs.68(12):2622-32.
20. Abbo L, Smith L, Pereyra M, Wyckoff M, Hooton TM. (2012) Nurse practitioners' attitudes, perceptions, and knowledge about antimicrobial stewardship. The Journal for Nurse Practitioners, **8**(5): 370-376
21. Adorka M, Dikokole M, Mitonga KH et al. (2013) Healthcare providers' attitudes and perceptions in infection diagnosis and antibiotic prescribing in public health institutions in Lesotho: a cross sectional survey. Afr Health Sci. **13**: 344-350.
22. Williams SJ., Halss AV, Moore, MV, Latter SE, Postle K, Leydon GM. (2017) General practitioner and nurse prescriber experiences of prescribing antibiotics for respiratory tract infections in UK primary care out-of-hours services (the UNITE study).*Journal of Antimicrobial Chemotherapy.* 73(3):795–803.
23. Courtenay M, Lim R, Castro-Sanchez E,et al (2018). Development of consensus-based national antimicrobial stewardship competencies for UK undergraduate healthcare professional education. J Hosp Infect.100(3):245-256. doi: 10.1016/j.jhin.2018.06.022. Epub 2018 Jun 30.
24. Courtenay M, Castro-Sanchez E, Gallagher R et al (2019). Development of consensus based national antimicrobial stewardship competencies for UK undergraduate nurse education. Journal of Hospital Infection, 103(3): 244-250.

25. Chater, A., Williams, J. & Courtenay, M. (2019). The prescribing needs of community practitioner nurse prescribers: A qualitative investigation using the Theoretical Domains Framework and COM-B. *Journal of Advanced Nursing.* doi: 10.1111/jan.14170

26. Chater, A. & Courtenay, M. (2019). Community nursing and antibiotic stewardship: the importance of communication and training. *British Journal of Community Nursing. 24(7),* 338-342.

27. Gilmartin-Thomas, J., Sleath, B., Cooper Bailey, S., Carpenter, D., Chater, A., MacAllister, C., Pyzik, O., Wayman, B., Annis, I. & Smith, F. (2019). Preparing pharmacy students to communicate effectively with adolescents. *International Journal of Pharmacy Practice.* doi: 10.1111/ijpp.12581

28. Jubraj, B., Barnett, N., Grimes, L., Varia, S., Chater, A., & Auyeung, V. (2016). Why we should understand the patient experience: Clinical empathy and Medicines Optimisation. *International Journal of Pharmacy Practice, 24(5), 367-370.* doi: 10.1111/ijpp.12268

29. Morgan, S. and Yoder, L.H. (2012) A concept analysis of patient-centred care. Journal of Holistic Nursing, 30:6-15.

30. Miller, W. R., & Rollnick, S. (2013). Motivational interviewing: Helping people change. New York, NY: Guilford Press.

31. Michie S, van Stralen MM, West R. (2011) The behaviour change wheel: a new method for characterising and designing behaviour change interventions. Implement Sci, **6**:42.doi:10.1186/1748-5908-6-42

32. IBM Corp. Released 2017. IBM SPSS Statistics for Windows, Version 25.0. Armonk, NY: IBM Corp.

33. Braun V & Clarke V (2006) Using thematic analysis in psychology. Qual Res Psychol, **3**:77–101.doi:10.1191/1478088706qp063oa

34. NVivo qualitative data analysis software. Version 10 QSR International Pty Ltd. 2014.

35. Creswell JW, Plano Clark VL (2007) *Designing and conducting mixed methods research*. Sage: Thousand Oaks.

36. Shenton A (2004) Strategies for ensuring trustworthiness in qualitative research projects. Education for Information, 22:63–7.

37. Sandelowski M. (1995) Triangles and crystals: on the geometry of qualitative research. Res Nurs Health, **18**:569–73. doi:10.1002/nur.4770180612

For peer review only

38. General Pharmaceutical Council (2019) Standards for the education and training of pharmacist independent prescribers. Accessed 10/10/19 https://www.pharmacyregulation.org/sites/default/files/document/standards-for-the-education-and-training-of-pharmacist-independent-prescribers-january-19.pdf

39. Royal Pharmaceutical Society (2016) A competency framework for all prescribers. Accessed 10/10/19 https://www.rpharms.com/Portals/0/RPS%20document%20library/Open%20access/Professional%20standards/Prescribing%20competency%20framework/prescribing-competency-framework.pdf

40. Courtenay M, Khanfer R, Harries-Huntly G, Deslandes R, Gillespie D, Hodson K, Morris G, Pritchard A and Williams E. (2017) Overview of the uptake and implementation of nonmedical prescribing in Wales: a national survey. BMJ Open 7:e015313. doi:10.1136/ bmjopen-2016-015313

Contributors MC made a substantial contribution to the conception and design of the work, interpretation of data and drafting of the work. RL made a substantial contribution to the design of the work, the acquisition, analysis and interpretation of data and drafting of the work. AC and DG made a substantial contribution to the design of the work and interpretation of data. KH made a substantial contribution to the conception of the work, participant recruitment and drafting of the work. RD, RF, NR and NT made a substantial contribution to the conception of the work and drafting of the work. All authors approved the final version to be published and agree to be accountable for all aspects of the work in ensuring that questions related to the accuracy or integrity of any part of the work are appropriately investigated and resolved.

Funding This work was supported by the Economic and Social Research Council (ESRC) Impact Acceleration Account (IAA).

Competing interests None declared.

Patient consent for publication Not required.

Ethics approval Ethical approval for the study has been provided by the School of Healthcare Sciences Research Governance and Ethics Committee, Cardiff University, UK.

For peer review only

Provenance and peer review Not commissioned; externally peer reviewed.

Data sharing statement No additional data available; that is, the dataset supporting the conclusions of this article is included within the article.

For peer review only

BMJ Open

A theory-based electronic learning intervention to support appropriate antibiotic prescribing by nurse and pharmacist independent prescribers: an acceptability and feasibility experimental study using mixed methods.

Journal:	*BMJ Open*
Manuscript ID	bmjopen-2019-036181.R1
Article Type:	Original research
Date Submitted by the Author:	24-Apr-2020

Complete List of Authors:	Lim, Rosemary; University of Reading School of Chemistry Food and Pharmacy, Reading School of Pharmacy<br>Courtenay, Molly; Cardiff University, Healthcare Sciences<br>Deslandes, Rhian; Cardiff University<br>Ferriday, Rebecca; Cardiff University<br>Gillespie, David; Centre for Trials Research, University of Cardiff ,<br>Hodson, Karen; Cardiff University<br>Reid, Nicholas; Public Health Wales,<br>Thomas, Neil; Cardiff University<br>Chater, Angel; University of Bedfordshire, Department of Sport Science and Physical

**Primary Subject Heading**:	General practice / Family practice
Secondary Subject Heading:	Respiratory medicine
Keywords:	EDUCATION & TRAINING (see Medical Education & Training), PRIMARY CARE, Respiratory infections < THORACIC MEDICINE

SCHOLARONE™
Manuscripts

I, the Submitting Author has the right to grant and does grant on behalf of all authors of the Work (as defined in the below author licence), an exclusive licence and/or a non-exclusive licence for contributions from authors who are: i) UK Crown employees; ii) where BMJ has agreed a CC-BY licence shall apply, and/or iii) in accordance with the terms applicable for US Federal Government officers or employees acting as part of their official duties; on a worldwide, perpetual, irrevocable, royalty-free basis to BMJ Publishing Group Ltd ("BMJ") its licensees and where the relevant Journal is co-owned by BMJ to the co-owners of the Journal, to publish the Work in this journal and any other BMJ products and to exploit all rights, as set out in our licence.

The Submitting Author accepts and understands that any supply made under these terms is made by BMJ to the Submitting Author unless you are acting as an employee on behalf of your employer or a postgraduate student of an affiliated institution which is paying any applicable article publishing charge ("APC") for Open Access articles. Where the Submitting Author wishes to make the Work available on an Open Access basis (and intends to pay the relevant APC), the terms of reuse of such Open Access shall be governed by a Creative Commons licence – details of these licences and which Creative Commons licence will apply to this Work are set out in our licence referred to above.

Other than as permitted in any relevant BMJ Author's Self Archiving Policies, I confirm this Work has not been accepted for publication elsewhere, is not being considered for publication elsewhere and does not duplicate material already published. I confirm all authors consent to publication of this Work and authorise the granting of this licence.

For peer review only

A theory-based electronic learning intervention to support appropriate antibiotic prescribing by nurse and pharmacist independent prescribers: an acceptability and feasibility experimental study using mixed methods.

Rosemary Lim, Associate Professor in Medication Use and Safety, Reading School of Pharmacy, University of Reading, PO Box 226, Whiteknights, Reading, Berkshire RG6 6AP, UK

Molly Courtenay, Professor of Health Sciences, School of Healthcare Sciences, Cardiff University, Cardiff, CF24 OAB, UK

Rhian Deslandes, Senior Lecturer, Cardiff School of Pharmacy and Pharmaceutical Sciences, Cardiff University, Cardiff, CF10 3NB, UK

Rebecca Ferriday, Learning Technology Manager, School of Healthcare Sciences,

Cardiff University, Cardiff, CF24 OAB, UK

David Gillespie, Deputy Director of Infection, Inflammation & Immunity Trials, Senior Research Fellow, Centre for Trials Research

Cardiff University, Cardiff, CF24 OAB, UK

Karen Hodson, Senior Lecturer, Cardiff School of Pharmacy and Pharmaceutical Sciences, Cardiff University, Cardiff, CF10 3NB, UK

Nicholas Reid, All Wales Consultant Antimicrobial Pharmacist, Healthcare Associated Infection, Antimicrobial Resistance & Prescribing Programme (HARP team),

Public Health Wales, Cardiff, CF10 4BZ, UK

Neil Thomas, Lecturer, School of Healthcare Sciences,

Cardiff University, Cardiff, CF24 OAB, UK

Angel Chater, Reader in Health Psychology and Behaviour Change

University of Bedfordshire, Bedford, MK419EA, UK, University College London School of Pharmacy, London, WC1H 9JP, UK

Corresponding author

Rosemary Lim, Email: r.h.m.lim@reading.ac.uk

For peer review only

Abstract

Objectives: To assess the acceptability and feasibility of using a theory-based electronic learning intervention designed to support appropriate antibiotic prescribing by nurse and pharmacist independent prescribers for patients presenting with common, acute, uncomplicated self-limiting respiratory tract infections (RTIs).

Design: Experimental with mixed methods; pre- and post-intervention online surveys and semi-structured interviews.

Setting: Primary care settings across the United Kingdom.

Participants: 11 nurse and 4 pharmacist prescribers.

Intervention: A theory-based brief interactive animation electronic learning activity comprised of a consultation scenario by a prescriber with an adult presenting with a common, acute, uncomplicated self-limiting RTI to support a 'no antibiotic prescribing strategy.'

Outcome measures: Recruitment, response and attrition rates were assessed. Overall usefulness of the intervention was assessed by analysing prescribers' self-reported confidence and knowledge in treating patients with RTIs before and after undertaking the intervention, and views on the relevance of the intervention to their work. Acceptability of the intervention was assessed in semi-structured interviews. The feasibility of data collection methods was assessed by recording the number of study components completed by prescribers.

Results: 15 prescribers (maximum sample size) consented and completed all four stages of the study. Prescribers reported high to very high levels of confidence and knowledge pre- and post-intervention, with slight post-intervention increases in communicating with patients and a slight reduction in building rapport. Qualitative findings supported quantitative findings; prescribers were reassured of their own practice which in turn increased their confidence and knowledge in consultations. The information in the intervention was not new to prescribers but was applicable and useful to consolidate learning and enable self-reflection. Completing the e-learning intervention was acceptable to prescribers.

For peer review only

Conclusions: It was feasible to conduct the study. The intervention was acceptable and useful to prescribers. Future work will add complex clinical content in the intervention before conducting a full trial.

Strengths and limitations of this study

- To our knowledge, this study was the first to examine the acceptability and feasibility of using an electronic, interactive, animation-based learning intervention to support appropriate antibiotic prescribing by nurse and pharmacist independent prescribers for patients presenting with common, acute, uncomplicated self-limiting respiratory tract infections.
- A mixed-methods approach allowed for validation of quantitative findings; interview findings enabled a richer picture to the contextual factors affecting the feasibility and acceptability of the intervention.
- The successful recruitment of nurse and pharmacist prescribers and completion of the intervention demonstrated the acceptability and feasibility of using the intervention amongst nurse prescribers.
- Prescribers were an opportunistic sample, generally more experienced and may therefore be biased towards appropriate prescribing.

Keywords: antibiotics, prescribing, nurse prescribers, pharmacist prescribers, electronic learning, antimicrobial stewardship

For peer review only

Introduction

Each year, antimicrobial resistant (AMR) infections cause approximately 700,000 deaths globally. By 2050 it has been predicted that this will rise to 10 million, combined with a cumulative cost of $100 trillion [1]. In the European Union and the European Economic Area, the figure is an estimated 33 110 deaths, 875 000 disability-adjusted life-years [2] and €1.5 billion in extra healthcare costs [3]. The inappropriate use of antimicrobials in humans is one of the leading drivers for the growth of AMR [4] and strategies to support appropriate antibiotic use are important. A key global strategy is careful stewardship of antibiotics [1]. Antimicrobial stewardship (AMS), comprises "a collection of co-ordinated interprofessional focused strategies to optimise antibiotic use by ensuring that every patient receives an antibiotic only when it is clinically indicated and then receives the appropriate antibiotic, at the right dose, duration and route of administration" [5].

Common, acute, uncomplicated self-limiting respiratory tract infections (RTIs) usually resolve spontaneously, with antibiotics in most cases, unlikely to offer clinical benefit [6]. These common conditions include acute otitis media (no otorrhoea i.e. discharge following an ear drum perforation and not a child under 2 years old with otitis media in both ears) [7], acute sore throat/acute pharyngitis/acute tonsillitis (FeverPAIN score 0 or 1 or Centor score 0, 1, 2) [8], acute sinusitis (symptoms lasting 10 days or less) [9] and acute cough (associated with an upper respiratory tract infection and acute bronchitis) [10]. Despite this, more than 60% of all prescriptions issued in the United Kingdom (UK) primary care are for RTIs [11, 12]. As well as contributing to the spread of resistance, their unnecessary use also puts patients at risk of side effects [13]. A global priority that has been recognised for some time is the need to conserve antibiotic sensitivity by managing RTIs without recourse to antibiotics and healthcare professionals who routinely prescribe antibiotics is a key target for interventions [13-16].

Multifaceted interventions that address barriers to change in specific healthcare settings involving active education strategies, feedback on antibiotic prescribing behaviour and seeking to improve prescribing for all as opposed to specific respiratory infections, tend

For peer review only

towards greater effectiveness in medical prescribers [17-18]. Comparable reductions in the utilisation of antibiotics by medical prescribers have been demonstrated using these strategies via electronic learning [19].

Appropriately qualified nurses and pharmacists in the UK can prescribe medicines independently and around 34,000 nurses and 8,000 pharmacists have independent prescribing capability [20]. Nurse and pharmacist prescribers frequently manage patients with RTIs and prescribe around 8% of all primary care antibiotics dispensed in England [21]. As compared with medical prescribers, a broader range of factors influence the prescribing behaviour of these professionals [22]. Factors include diagnostic uncertainty and the patient's clinical condition [22-25], the expectations of patients for an antibiotic [22-23, 25], relationships with other prescribers and knowledge of current guidelines [22-23, 26]. Interventions are available to support the various AMS activities that healthcare professionals are involved in [27-28] however, no interventions exist to specifically support appropriate antibiotic prescribing behaviour by nurse and pharmacist independent prescribers. We developed a theory-based electronic learning intervention that aimed to support a 'no antibiotic prescribing strategy' by nurse and pharmacist independent prescribers for common, acute, uncomplicated self-limiting RTIs to reflect national (NICE) prescribing guidance. The aim of this study was to assess the acceptability and feasibility of implementing this e-learning intervention in UK general practice.

Method

Ethical considerations

The School of Healthcare Sciences Research Governance and Ethics Committee, Cardiff University gave ethical approval for the conduct of this study (reference: 427REC). All participants gave written informed consent before participating in the study.

Design

For peer review only

A mixed-methods study design was used: participants completed an online pre- and post-intervention survey, participated in the intervention and a semi-structured interview with a researcher.

Participant recruitment

The inclusion criteria for participants were nurse and pharmacist non-medical independent prescribers (hereafter described as prescribers) who managed patients with RTIs in a primary care setting in the UK. Recruitment took place during November to December 2018 via several routes until a maximum of 15 nurse and pharmacist prescribers in total had been recruited; a sample size expected to enable qualitative data saturation [20]: 1. MC emailed prescribers (4 pharmacists and 17 nurses) who consented to be contacted after taking part in previous research led by MC [22], 2. KH and RL approached key contacts within their existing prescriber networks who sent out information about the study via email (approximately 195 prescribers and nurses combined). MC or RL emailed the participant information sheet and consent form to interested prescribers and encouraged them to ask any questions they may have about the study prior to making an informed choice about participating in the study. Participants gave written consent prior to taking part in the study.

Intervention

The theory-based intervention comprised a five-minute, interactive, animated scenario of a consultation by a prescriber with an adult presenting with a common, acute, uncomplicated self-limiting RTI. The development of the intervention is published elsewhere [20] and draws from previous work with nurse prescribers [29, 30] and pharmacists [31, 32]. The intervention was designed using the Behaviour Change Wheel (BCW) [33]. The BCW three-stage, eight-step approach was used, which included the development of the intervention content taking into consideration Capability, Opportunity and Motivation-Behaviour (COM-B) and Theoretical Domains Framework (TDF). The COM-B was used to create a behavioural diagnosis i.e. what needs to change; and the TDF identified relevant psychosocial drivers of the behaviour. The intervention functions, ‘education’, ‘training’ and ‘modelling’ were

For peer review only

identified the most appropriate for changing AMR prescribing behaviour, using the COM-B mapping from the BCW. The development of the e-learning activity was underpinned by 'gamification' as a teaching approach, specifically using a 'spot the difference' format animation. There is increasing evidence of improved knowledge among healthcare professional on sepsis management [34] and antibiotic use [35], following usage of online games despite previous inconclusive evidence of the utility of educational games for health professionals [36].

Prescribers were sent a weblink to the intervention that was accessible on any internet-enabled device. They were asked to engage with the e-learning activity by watching and answering questions posed within the activity. The intervention comprised two scenarios with the first depicting a consultation by a prescriber to reach a no antibiotic prescribing decision whereby patient-centred approaches [37] such as holistic care, individualised care, respectful care and empowering care, to reach a prescribing decision were absent. The overall patient experience and satisfaction was poor; the patient was not confident in the treatment decision made in the consultation. This is followed by a second scenario where a prescriber used a patient-centred motivational interviewing style [38] to reach a no antibiotic prescribing decision in the consultation. In this scenario, the patient left the consultation satisfied and confident with the treatment received. To facilitate active learning, prescribers were explicitly asked to 'spot the difference' between the two scenarios by answering a range of open and closed questions that were incorporated in this second scenario. These questions focused on the different approach the second prescriber undertook to consult with the patient.

Measures and data collection

The study was conducted in four stages in the following order: (1) pre-intervention online questionnaire, (2) e-learning intervention, (3) post-intervention online questionnaire and (4) semi-structured telephone interviews. Overall usefulness of the intervention was assessed by analysing prescribers' self-reported confidence and knowledge in treating patients with RTIs before and after undertaking the intervention, and views on the relevance of the intervention to their work. Acceptability of the intervention was assessed in semi-structured

For peer review only

interviews. The feasibility of data collection methods was assessed by recording the number of study components completed by prescribers. Responses to open and closed questions in the e-learning intervention were not collated and analysed because the aim of the study was to assess the acceptability and feasibility of implementing the intervention.

Pre-post intervention online questionnaires

Prescribers were sent a link and completed an online questionnaire before and immediately after completing the intervention, aimed to assess their perceptions of the impact of the intervention. The questionnaires were developed based on findings from our previous work [20]. The pre-intervention questionnaire assessed prescribers' knowledge and confidence prescribing antibiotics for patients presenting with RTIs. Prescribers were asked to rate their responses on six items using a 5-point Likert scale (Strongly Disagree to Strongly Agree): (1) gain information on patient expectations, (2) support patients, (3) build rapport, (4) communicate effectively, (5) see and examine different viewpoints, (6) ensure patients both understand and are happy with the prescribing decision. The post-intervention questionnaire contained the same questions as the pre-intervention questionnaire, and six additional questions about the usefulness of the intervention: (1) whether the information was known to participants, (2) its applicability to practice, (3) whether the intervention would be useful to them as prescribers, (4) whether it makes them feel more comfortable when speaking with patients with RTIs, (5) if it encourages participants to consider how they would apply the information to practice and (6) think differently. In both questionnaires, demographic details were also collected: type of prescriber (i.e. nurse or pharmacist), length of time qualified as a prescriber, time in current post, clinical setting and length of consultation time.

Semi-structured interviews

Following completion of the intervention and the post-intervention questionnaire, prescribers were invited to take part in a semi-structured telephone interview to understand prescribers' experiences of using the intervention. The interview took place

For peer review only - http://bmjopen.bmj.com/site/about/guidelines.xhtml

within 1-2 weeks post intervention to ensure retention of information. Interviews (see Box 1 for the interview schedule) explored each component of the COM-B model, the hub of the Behaviour Change Wheel [31] to create a behavioural diagnosis to understand Capability (e.g. knowledge, skills), Opportunity (e.g. social influence, environment) and Motivation (e.g. beliefs about consequences and emotional responses). Demographic details such as job title, practice setting, years qualified as a prescriber and approximate frequency of RTI consultations and antibiotic prescribing were also collected in the interview. All interviews were digitally recorded and then transcribed verbatim with identifying information removed.

Box 1 Interview schedule

1) Capability

Examples of prompts:

- Did the learning resource increase your knowledge and skills about prescribing antibiotics for respiratory tract infections?
- (if yes) Can you tell me in what ways?
- (if no) Can you tell me why this was the case for you?
- Did it increase your awareness of potential solutions to any difficulties you may have experienced managing the consultations of patients with RTIs?
- (if yes) Can you tell me in what ways?
- (if no) Can you tell me why this was the case for you?
- How do you think your practice will change based on the training resource?

2) Opportunity (relating to the resource and also behaviour to be changed)

Examples of prompts:

- How do you think this resource would change the norms of practice? Or the Practice of others. How will this fit in to current practice? (ie in terms of time)
- How did the resource address any gaps in your prescribing practice?
- Is the resource an acceptable/feasible delivery method that can be integrated into daily practice?

3) Motivation

Examples of prompts:

- In what way does the resource provide a means by which you can reflect on and develop your practice?
- How do you perceive that this resource could improve prescribing practice generally?
- How did it make you feel about their current and future practice?

For peer review only

4) Other questions

- What stood out for you the most, in the learning resource? E.g. was it the poster – was it that she came to greet the patient etc…
- How can the learning resource be improved? Was there anything that you would change for future training?

Data analysis

Quantitative data were collected prior to the qualitative interviews and both datasets were analysed separately but within the same time frame. Quantitative findings informed the qualitative interviews and the qualitative findings explained in more detail, the quantitative findings. An iterative process of comparing both the quantitative and qualitative datasets allowed for data triangulation and confirmed the accuracy of findings in the interviews, providing further insight to the phenomenon under study [39, 40].

Pre-post intervention online questionnaires

Data were analysed using SPSS version 25 [41]. Descriptive statistics were used to characterise prescribers (frequencies and percentages), prescribers' knowledge and confidence in prescribing antibiotics for patients presenting with RTIs (mean, SD) and their views on the usefulness of the intervention (mean, SD).

Semi-structured interviews

Interviews were analysed using inductive thematic analysis [42]. Coding and categorising of data was conducted by RL using NVivo 10 [43]. Themes were then identified reviewed with a second researcher (MC) and any differences in interpretation were resolved through discussion to increase the trustworthiness of research data [44, 45].

For peer review only

Patient and public involvement

Patients and the public were not involved in the development of the research question, study design, recruitment and conduct of the study.

Results

A total of 15 of the 216 prescribers approached (7%) responded to the study invitation, consented and completed all four stages of the study between October and December 2018. The maximum number of participants targeted for the study was reached. Table 1 shows the demographic data of prescribers. Most prescribers worked in general practice (*n*=10, 67%) and had been qualified for at least two years (*n*=12, 80%). Except for prescribers in the community care setting, most prescribers worked within 10 to 30 minutes patient appointment times. Interviews with prescribers lasted between 7 and 28 minutes (mean = 16 minutes).

Table 1: Demographic data of participants (n=15)

**Type of non-medical prescriber**	
Nurse	11 (73.3%)
Pharmacist	4 (27.7%)
**Time qualified as prescriber**	
13months to 2 years	3 (20.0%) *
3-5 years	5 (33.3%)
6-10 years	2 (13.3%)
>10 years	5 (33.3%)
**Time in post**	
13 months to 2 years	4 (26.7%)
3-5 years	9 (60.0%)
6-10 years	0 (0.0%)
>10 years	2 (13.3%)
**Type of clinical setting and length of patient appointments**	
Community care (45-90 minutes)	2 (13.3%)
General practice (10-15 minutes)	10 (66.7%)
Out of hours (10-20 minutes)	1 (6.7%)
General practice and out of hours (10-30 minutes)	1 (6.7%)
Urgent care (15 minutes)	1 (6.7%)

For peer review only

Note: * Two of the three participants were pharmacist. # All pharmacists worked in general practice and had 15 minutes appointment times.

Impact of intervention on prescribing practice

Table 2 shows prescribers' scores relating to their confidence in managing patients presenting with RTIs pre- and post-intervention. High to very high levels of confidence were reported for all statements both pre- and post-intervention. There was an increase in confidence levels in statements 1.2 (supporting patients understand health information given), 1.4 (skills to communicate with patients) and 1.5 (skills to help patients see and examine different viewpoints). Participants scored very highly for statement 1.3 (building rapport with patients) both pre- and post-intervention but there was a slight reduction in confidence post-intervention. Prescribers' confidence in their own ability to gain health-related information and that patients understand and are happy with their prescribing decision stayed the same.

Table 2: Confidence in treatment management of patients with RTI pre and post-intervention.

	Pre-intervention		Post-intervention		Mean change
**Statement**	**Min, Max**	**Mean (SD)**	**Min, Max**	**Mean (SD)**	
1.1 Confidence in gaining health-related information	3, 5	4.20 (0.68)	3, 5	4.20 (0.56)	0
1.2 Confidence in supporting patients understand health information given	4, 4	4.00 (0.00)	4, 5	4.27 (0.46)	0.27
1.3 Confidence in building rapport with patients	4, 5	4.53 (0.52)	4, 5	4.47 (0.52)	-0.06
1.4 Confidence in skills to communicate with patients	4, 5	4.53 (0.52)	3, 5	4.60 (0.63)	0.07
1.5 Confidence in skills to help patients see and examine different viewpoints	3, 5	3.87 (0.74)	3, 5	4.13 (0.64)	0.26
1.6 Confidence that patients understand and happy with prescribing decision	2, 5	4.07 (0.80)	3, 5	4.07 (0.46)	0

SD = standard deviation

For peer review only

Interviews with prescribers supported quantitative findings and revealed that watching and learning 'good practice', as shown in the intervention, reassured them of their own practice. It appeared to increase their confidence in their current practice and in refusing to prescribe antibiotics, when appropriate.

> *"So, when I watched it, I was thinking, well, yeah, I do, it reinforced what I was doing was right… and I just, you know, keep doing that." Nurse 10*

> *"It's given me more confidence in my approach really, to managing those patients with the respiratory tract infections." Pharmacist 4*

Usefulness of intervention

Table 3 shows prescribers' level of agreement with statements relating to the usefulness of the intervention. Responses were recorded on a 5-point Likert scale: 1= strongly disagree, 2 = somewhat disagree, 3 = neither agree nor disagree, 4 = somewhat strongly agree and 5 = strongly agree. Although prescribers disagreed that the information in the intervention was new to them, they agreed that the intervention was applicable and would be useful to them as prescribers.

Table 3: Usefulness of intervention from post-intervention questionnaire

Statement	Minimum	Maximum	Mean	Std. Deviation
2.1 The information was mostly new to me	1	2	1.33	0.49
2.2 The intervention was applicable to my practice	2	5	4.60	0.83
2.3 The intervention will be useful to me as a prescriber	3	5	4.20	0.78
2.4 The intervention has made me feel more comfortable speaking with patients with RTIs	2	4	3.13	0.83
2.5 The intervention has encouraged me to consider how I would apply the information in my practice	2	5	3.87	0.99
2.6 The intervention has encouraged me to think differently	1	4	3.07	1.10

For peer review only - http://bmjopen.bmj.com/site/about/guidelines.xhtml

Qualitative data was consistent with the questionnaire data; the majority of prescribers did not think that they had acquired new knowledge or skills from the e-learning intervention because they were largely an experienced group of practitioners. For most prescribers, however, the intervention was useful in helping them refresh their memories, consolidate learning and prompted self-reflection on prescribing for patients presenting with respiratory tract infections.

"I've been doing it quite a long time now and, you know, especially in my respiratory clinic, so I suppose, some of it was just reinforcing what I already sort of knew, but then that's good as well. It's still teaching, isn't it? It's still learning?" Nurse 6

"Yeah, I think it's about, you know, I like to do snippets of education and I sell it as… and this is very much this. It's the cement between the bricks. Your day's bricks and this is just a little filler that builds it all together." Nurse 3

"I think to… to quite a big extent, I've probably been prescribing more than I needed to…I often deal with the very frail and elderly. I often deal with the very frail and elderly. And urine infections the whole time… in the past, I have really thought, oh, crikey, they're old, they must have antibiotics. But actually, that's not necessarily a good thing." Nurse 11

Some learnt other methods of communicating with patients, for example, the use of additional information such as posters and leaflets and scheduling follow-up appointments after the consultation as a 'safety net'.

"I would never really have thought to... to contact the patients... I'll think about doing that in the future. And who... who are worried, um, about not prescribing antibiotics for them, um, I think that... that would ease the pressure from it." Pharmacist 4

For peer review only

Despite claiming not learning new knowledge, some prescribers said they intended to make changes to their practice specifically around involving the patient more in decision-making and directing patients to relevant information resources. A few prescribers claimed to have already changed their practice, for example, in considering different approaches to arrive at treatment decisions and using different ways of communicating with patients.

> *"I'll be sort of more vigilant in making sure that, you know, the patient understands why, I've, you know, decided to go with a certain treatment and sort of try and get the patient to engage and agree with that decision…So they're involved within the decision and I think that kind of gives them a better understanding." Pharmacist 1*

> *"…having a simple resource that I can always apply to my consultations…was actually really helpful for that and I've used it in that context a number of times with different patients. With palpable different outcomes really, I think, you know. I have, you know, embarked on the education, rather than prescription approach, in… in consequence to doing it, so it has been good for that." Nurse 4*

> *"I began to think much more clearly in the terms of, give me a reason to give them antibiotics. So I think that was a little bit of a theme change for me. Because I'd always thought, oh, I don't want to upset… well, I don't like upsetting people. I don't. I like people to go away feeling as if I've listened to them… And they maybe have not got what they came in to look for, but they're satisfied." Nurse 11*

Acceptability of the intervention

Design and content of the intervention

Prescribers said they liked the use of e-learning because there was flexibility of where and when learning can take place. The use of 'cartoon' characters and scenes generated mixed views. The scenario-based learning approach was well-received; the scenario was realistic and memorable.

For peer review only

"And that will… stay with me. You know, when I think about it, that's the vision that I have, is this person typing really fast. It's just… it was just funny. I just thought, God, that's all of us. We're just typing really fast, thinking come on! Hurry up, hurry up!" Nurse 1

"I liked the scenario around it as that seemed real. And it's… it's reassuring actually those conversations that were had in the video, which we all know we all have…It kind of just said, you know, we do have these difficult things. You know, patients sometimes come in, they want antibiotics and they… you know, they perceive the need for them, but it was nice to see that we can say, you don't need them, it's okay." Nurse 3

Prescribers said the messages presented in the e-learning intervention were appropriate, relevant, easy to understand and consistent with their own previous learning and practice. Some messages that stood out included managing patient expectations by using posters in the waiting room and providing self-care advice.

"I know and I know that, um, my GP colleagues also, we all struggle with the patient expectation of antibiotics, so I liked very much the idea of managing expectations of having… having the posters in the waiting room, of having the literature available." Nurse 2

Some prescribers highlighted the use of effective communication and observational skills in the intervention to be valuable aspects of the intervention.

"…you know when she tells the, er, patient the second time round why she's not giving a… why she doesn't need antibiotics… I think that that part really stood out for me. Because I think it was explained clearly, it was precise, um, it wasn't too long, it wasn't too short. And it was just… just right and I think that the level of language used. What was… I really liked it. I think it was spot on really." Pharmacist 1

For peer review only

Completing the intervention

All the prescribers said it was quick (approximately 5 to 20 minutes) to complete the intervention. Around half of the prescribers said they completed the intervention at work for example during their lunch break, in between appointments whilst the rest completed the intervention in their own time. Despite this, prescribers agreed that the intervention could be completed during work time because it was a short learning session.

Suggestions for improving the e-learning intervention

Perceived and potential demographic of learners

Most prescribers said that the content in the e-learning intervention was pitched at a lower level of experience and suggested additional groups of people who may benefit from it such as GPs, GP trainees, new medical and NMPs, undergraduate healthcare students and the public.

> *"I thought at times that the level was a little bit lower. I thought the level being pitched was a little bit low and I don't know if that was intentional." Nurse 3*

> *"I suppose what the content of the… the little video was, was fairly sort of low level, in terms of antimicrobial prescribing. I suppose it's the sort of thing that I would expect maybe most people prescribing in that environment to know." Nurse 9*

Access

A few prescribers reported experiencing problems accessing the intervention at work due to an NHS firewall. They circumvented the problem by using their own personal device.

Overall presentation

A few prescribers suggested using a more diverse set of characters e.g. gender, race, age, and a more realistic layout of a consulting room.

For peer review only

Content

Other suggestions included adding learning outcomes to set learners' expectations, providing additional information such as an estimated time to complete the intervention, context to the scenario and patient history, more information about aspects shown in the learning intervention, for example, relevance and information about the poster, physical examinations, highlight key messages and including questions on self-reflection within the intervention. There was also a suggestion to include subtitles to the scene. Some prescribers suggested adding scenarios with different challenges for example, a difficult patient, different groups of patients, patient re-visits. To accommodate different learner requirements, some prescribers suggested including different levels of clinical content such as the most up-to-date clinical guidelines and additional reading materials.

For peer review only

Discussion

Completing the e-learning intervention was acceptable to prescribers. It was also feasible to collect study data in four separate stages. Overall, prescribers reported positive views about the usefulness of the intervention. Prescribers reported high levels of confidence in managing patients with RTIs both pre- and post-intervention. Whilst recruitment of prescribers to the study was successful, the recruitment of pharmacist prescribers was challenging. Nationally, there are fewer pharmacist prescribers compared to nurse prescribers [21] and further consideration to sample size will be needed to inform the next stage of this study.

The intervention component of the study was short, around 5 minutes, and all prescribers were able to complete the intervention at a time and place that suited them. Although some prescribers chose to complete the intervention in their personal time, all agreed that it was feasible to complete the intervention as part of their day-to-day work suggesting the acceptability of using the intervention as part of their training. There was overall support for using an e-learning mode of delivery and this was consistent with the suggestion made by a similar group of prescribers [15] however, there were reported issues with accessing the intervention on NHS computers. The next iteration of the intervention will need to consider

wider access issues if the learning were to take place as part of routine training in the future.

Prescribers reported high to very high levels of confidence for all statements both pre- and post-intervention and the finding was consistent with the qualitative dataset. The findings could be explained by the demographics of the study population – a group of prescribers who were largely very experienced in their role; nurse and pharmacist prescribers have often undertaken postgraduate specialist training and although many nurse prescribers have undertaken specialist qualifications, they have not all done so and only allowed to prescribe within their area of competence [46-48]. Although there were small increases in confidence levels related to skills relating to communicating with patients and a slight reduction in building rapport with patients, it was not possible to make generalisations due to the small sample size. Prescribers were also an opportunistic sample, were generally more experienced and may therefore be biased towards appropriate prescribing. Although interviews took place as soon as possible after prescribers had completed the intervention and post-intervention questionnaire, these typically took place after 2 weeks and this time lag could affect recall of their experiences of undertaking the intervention.

It is however worth noting that prescribers reported that the intervention enabled self-reflection and the slight reduction in confidence in building rapport with patients could be the result of prescribers' reflection of their practice. Regarding usefulness of the intervention, prescribers scored low on aspects relating to learning new information in the intervention. But, prescribers generally considered the intervention to be useful because it provided the opportunity to remind themselves on the topic, enabled self-reflection and change practice. Reflecting on one's professional practice is a key requirement for pharmacist and nurse revalidation [49, 50] and is an interesting outcome that was not expected or measured in this study. The brief and easily accessible intervention used in this study is similar to the learning that healthcare staff are increasingly accessing e.g. 'Espresso' sessions held at workplaces, suggesting the potential for widespread adoption.

Although the development of the intervention was directly informed by research conducted with prescribers with similar demographic characteristics as those in this study and underpinned by psychological and pedagogic theories, there were aspects to the intervention that requires further refinement specifically the level of difficulty of the clinical

For peer review only

content. Intervention development, testing and evaluation is an iterative process and should be based on a user-centred approach [51, 52]. The next iteration of the e-learning intervention will focus on the addition of complex clinical content, possibly multiple activities and testing focused on the appropriateness of the clinical content.

Strengths and limitations

To our knowledge, this study was the first to examine the acceptability and feasibility of using an electronic, interactive, animation-based learning intervention to support appropriate antibiotic prescribing by nurse and pharmacist independent prescribers for patients presenting with common, acute, uncomplicated self-limiting respiratory tract infections. A mixed-methods approach allowed for validation of quantitative findings; interview findings enabled a richer picture to the contextual factors affecting the feasibility and acceptability of the intervention. The successful recruitment of nurse and pharmacist prescribers and completion of the intervention demonstrated the acceptability and feasibility of using the intervention amongst nurse prescribers.

A small sample was recruited to determine the acceptability and feasibility of using the intervention. Therefore, findings will need to be interpreted with caution. Prescribers were also an opportunistic sample, were generally more experienced and may therefore be biased towards appropriate prescribing. Although interviews took place as soon as possible after prescribers had completed the intervention and post-intervention questionnaire, these typically took place after 2 weeks and this time lag could affect recall of their experiences of undertaking the intervention.

Conclusions

The study showed that it was feasible to conduct the study and the intervention was acceptable and useful to prescribers. Suggestions to improve the usefulness of the intervention focused on the clinical content rather than its delivery. Future work will consider the addition of complex clinical content in the intervention before considering a full trial.

For peer review only

Acknowledgements

We would like to thank all the prescribers who took part in the study, and network contacts who helped to disseminate information about the study.

References

1. Her Majesty's (HM) Government (2019). Tackling antimicrobial resistance 2019-2024 – The UK's five-year national action plan. Accessed 11 February 2019
2. Cassini A, Högberg LD, Plachouras D, Quattrocchi A, Hoxha A, Simonsen GS, Colomb-Cotinat M, Kretzschmar ME, Devleesschauwer B, Cecchini M, Ouakrim DA, Oliveira TC, Struelens MJ, Suetens C, Monnet DL, and the Burden of AMR Collaborative Group. (2019) Attributable deaths and disability-adjusted life-years caused by infections with antibiotic-resistant bacteria in the EU and the European Economic Area in 2015: a population-level modelling analysis. Lancet Infect Dis 19: 56–66 http://dx.doi.org/10.1016/ S1473-3099(18)30605-4
3. European Commission - Fact Sheet. New EU action plan on antimicrobial resistance – questions and answers: Brussels 2017.
4. Holmes AH, Moore LSP, Sundsfjord A, et al.Understanding the mechanisms and drivers of antimicrobial resistance. Lancet. 2015;6736: Available at: http://linkinghub.elsevier.com/retrieve/pii/S0140673615004730
5. Manning ML, Pfeiffer J, Larson EL. Combating antibiotic resistance: The role of nursing in antibiotic stewardship. Am J Infect Control 2015; 44(12):1454-1457
6. NICE. Short clinical guidelines technical team. respiratory tract infections – antibiotic prescribing. prescribing of antibiotics for self-limiting respiratory tract infections in adults and children in primary care. London National Institute for Health and Clinical Excellence; 2008. https://www.nice.org.uk/guidance/cg69
7. NICE (2018) Otitis media (acute): antimicrobial prescribing. https://www.nice.org.uk/guidance/ng91/chapter/Recommendations#managing-acute-otitis-media

For peer review only

8. NICE (2018) Sore throat (acute): antimicrobial prescribing https://www.nice.org.uk/guidance/ng84/chapter/Recommendations#managing-acute-sore-throat
9. NICE (2017) Sinusitis (acute): antimicrobial prescribing https://www.nice.org.uk/guidance/ng79/chapter/Recommendations#managing-acute-sinusitis
10. NICE (2019) Cough (acute): antimicrobial prescribing https://www.nice.org.uk/guidance/ng120/chapter/Recommendations#treatment
11. Gulliford MC, van Staa T, Dregan A, McDermott L, McCann G, Ashworth M, Charlton J, Little P, Moore MV, Yardley L (2014) Electronic health records for intervention research: a cluster randomized trial to reduce antibiotic prescribing in primary care (eCRT study). Ann Fam Med. 12(4):344-51. doi: 10.1370/afm.1659
12. Public Health England (2014) English Surveillance Programme for Antimicrobial Utilisation and Resistance *(ESPAUR) Report 2010 to 2013.*
13. Goossens H, Ferech M, Vander Stichele R, *et al.* (2005)Outpatient antibiotic use in Europe and association with resistance: a cross-national database study. Lancet, **365**:579-87.
14. Costelloe C, Metcalfe C, Lovering A, *et* al. (2010) Effect of antibiotic prescribing in primary care on antimicrobial resistance in individual patients: Systematic review and meta-analysis. BMJ, 340:c2096.
15. Sanders S, Glasziou PP, Del Mar CB, Rovers MM. Antibiotics for acute otitis media in children. Cochrane Database of Systematic Reviews 2004, Issue 1. Art. No.: CD000219. DOI: 10.1002/14651858.CD000219.pub2
16. WHO. Antimicrobial resistance Fact sheet No. 194. 2016. http://www.who.int/mediacentre/factsheets/fs194/en/ World Health Organization. Global action plan on antimicrobial resistance, 2015
17. Arnold SR, Straus SE. (2005) Interventions to improve antibiotic prescribing practices in ambulatory care. Cochrane Database Syst Rev. Issue 4. Art. No.: CD003539. DOI: 10.1002/14651858.CD003539.pub2
18. Ranji, S,Steinman, M, Shojania, K.,Gonzales, R., (2008) Interventions to Reduce Unnecessary Antibiotic Prescribing: A Systematic Review and Quantitative Analysis

For peer review only

Medical Care, 46(8): 847-862 doi: 10.1097/MLR.0b013e318178eabd

018; bjgp18X697889. DOI: https://doi.org/10.3399/bjgp18X697889

19. Little P, Stuart B, Francis N, Douglas E, Tonkin-Crine S, Anthierens S, et al. (2013) Effects of internet-based training on antibiotic prescribing rates for acute respiratory-tract infections: a multinational, cluster, randomised, factorial, controlled trial. Lancet, 382:1175–82.
20. Courtenay M, Lim R, Deslandes R, et al. (2019) Theory-based electronic learning intervention to support appropriate antibiotic prescribing by nurses and pharmacists: intervention development and feasibility study protocol. BMJ Open, 9:e028326. doi:10.1136/ bmjopen-2018-028326
21. Courtenay M, Gillespie D, Lim, R. (2017) Patterns of dispensed non-medical prescriber prescriptions for antibiotics in primary care across England: a retrospective analysis. Journal of Antimicrobial Chemotherapy. dkx230, https://doi.org/10.1093/jac/dkx230
22. Courtenay M, Rowbotham S, Lim R, et al. (2019).Examining influences on antibiotic prescribing by nurse and pharmacist prescribers: a qualitative study using the Theoretical Domains Framework and COM-B. BMJ Open;9:e029177. doi:10.1136/ bmjopen-2019-029177
23. Rowbotham S, Chisholm A, Moschogianis S, Chew-Graham C, Cordingley L, Wearden A, Peters S. (2012) Challenges to nurse prescribers of a no-antibiotic prescribing strategy for managing self-limiting respiratory tract infections. J Adv Nurs.68(12):2622-32.
24. Abbo L, Smith L, Pereyra M, Wyckoff M, Hooton TM. (2012) Nurse practitioners' attitudes, perceptions, and knowledge about antimicrobial stewardship. The Journal for Nurse Practitioners, **8**(5): 370-376
25. Adorka M, Dikokole M, Mitonga KH et al. (2013) Healthcare providers' attitudes and perceptions in infection diagnosis and antibiotic prescribing in public health institutions in Lesotho: a cross sectional survey. Afr Health Sci. **13**: 344-350.
26. Williams SJ., Halss AV, Moore, MV, Latter SE, Postle K, Leydon GM. (2017) General practitioner and nurse prescriber experiences of prescribing antibiotics for respiratory tract infections in UK primary care out-of-hours services (the UNITE study).*Journal of Antimicrobial Chemotherapy.* 73(3):795–803.

For peer review only

27. Courtenay M, Lim R, Castro-Sanchez E,et al (2018). Development of consensus-based national antimicrobial stewardship competencies for UK undergraduate healthcare professional education. J Hosp Infect.100(3):245-256. doi: 10.1016/j.jhin.2018.06.022. Epub 2018 Jun 30.
28. Courtenay M, Castro-Sanchez E, Gallagher R et al (2019). Development of consensus based national antimicrobial stewardship competencies for UK undergraduate nurse education. Journal of Hospital Infection, 103(3): 244-250.
29. Chater, A., Williams, J. & Courtenay, M. (2019). The prescribing needs of community practitioner nurse prescribers: A qualitative investigation using the Theoretical Domains Framework and COM-B. *Journal of Advanced Nursing.* doi: 10.1111/jan.14170
30. Chater, A. & Courtenay, M. (2019). Community nursing and antibiotic stewardship: the importance of communication and training. *British Journal of Community Nursing. 24(7),* 338-342.
31. Gilmartin-Thomas, J., Sleath, B., Cooper Bailey, S., Carpenter, D., Chater, A., MacAllister, C., Pyzik, O., Wayman, B., Annis, I. & Smith, F. (2019). Preparing pharmacy students to communicate effectively with adolescents. *International Journal of Pharmacy Practice.* doi: 10.1111/ijpp.12581
32. Jubraj, B., Barnett, N., Grimes, L., Varia, S., Chater, A., & Auyeung, V. (2016). Why we should understand the patient experience: Clinical empathy and Medicines Optimisation. *International Journal of Pharmacy Practice, 24(5), 367-370.* doi: 10.1111/ijpp.12268
33. Michie S, Atkins L, West R. (2014) The behaviour change wheel: a guide to designing interventions. London: Silverback Publishing.
34. Evans KH, Daines W, Tsui J, et al. (2015) Septris: a novel, mobile, online, simulation game that improves sepsis recognition and management. Acad Med, 90:180–4.
35. Ferrer R, Martínez ML, Gomà G, et al. (2018) Improved empirical antibiotic treatment of sepsis after an educational intervention: the ABISS-Edusepsis study. Crit Care 22(1):167.
36. Akl EA, Kairouz VF, Sackett KM, et al. (2013) Educational games for health professionals. Cochrane Database Syst Rev, CD006411.
37. Morgan, S. and Yoder, L.H. (2012) A concept analysis of patient-centred care. Journal of Holistic Nursing, 30:6-15.

For peer review only

38. Miller, W. R., & Rollnick, S. (2013). Motivational interviewing: Helping people change. New York, NY: Guilford Press.

39. Michie S, van Stralen MM, West R. (2011) The behaviour change wheel: a new method for characterising and designing behaviour change interventions. Implement Sci, **6**:42.doi:10.1186/1748-5908-6-42

40. Creswell JW, Plano Clark VL (2007) *Designing and conducting mixed methods research*. Sage: Thousand Oaks.

41. IBM Corp. Released 2017. IBM SPSS Statistics for Windows, Version 25.0. Armonk, NY: IBM Corp.

42. Braun V & Clarke V (2006) Using thematic analysis in psychology. Qual Res Psychol, **3**:77–101.doi:10.1191/1478088706qp063oa

43. NVivo qualitative data analysis software. Version 10 QSR International Pty Ltd. 2014.

44. Shenton A (2004) Strategies for ensuring trustworthiness in qualitative research projects. Education for Information, 22:63–7.

45. Sandelowski M. (1995) Triangles and crystals: on the geometry of qualitative research. Res Nurs Health, **18**:569–73. doi:10.1002/nur.4770180612

46. General Pharmaceutical Council (2019) Standards for the education and training of pharmacist independent prescribers. Accessed 10/10/19 https://www.pharmacyregulation.org/sites/default/files/document/standards-for-the-education-and-training-of-pharmacist-independent-prescribers-january-19.pdf

47. Royal Pharmaceutical Society (2016) A competency framework for all prescribers. Accessed 10/10/19 https://www.rpharms.com/Portals/0/RPS%20document%20library/Open%20access/Professional%20standards/Prescribing%20competency%20framework/prescribing-competency-framework.pdf

48. Courtenay M, Khanfer R, Harries-Huntly G, Deslandes R, Gillespie D, Hodson K, Morris G, Pritchard A and Williams E. (2017) Overview of the uptake and implementation of nonmedical prescribing in Wales: a national survey. BMJ Open 7:e015313. doi:10.1136/ bmjopen-2016-015313

49. General Pharmaceutical Council (2018) Revalidation Framework. https://www.pharmacyregulation.org/sites/default/files/document/gphc_revalidation_framework_january_2018.pdf

For peer review only

50. Nursing and Midwifery Council (2019) How to revalidate with the NMC: requirements for renewing your registration. https://www.nmc.org.uk/globalassets/sitedocuments/revalidation/how-to-revalidate-booklet.pdf

51. Michie S, Yardley L, West R, et al. (2017) Developing and evaluating digital interventions to promote behavior change in health and health care: recommendations resulting from an international workshop. J Med Internet Res, 19:e232.

52. Murray E, Hekler EB, Andersson G, et al. (2016) Evaluating digital health interventions: key questions and approaches. Am J Prev Med. 51:843–51.

Contributors MC made a substantial contribution to the conception and design of the work, interpretation of data and drafting of the work. RL made a substantial contribution to the design of the work, the acquisition, analysis and interpretation of data and drafting of the work. AC and DG made a substantial contribution to the design of the work and interpretation of data. KH made a substantial contribution to the conception of the work, participant recruitment and drafting of the work. RD, RF, NR and NT made a substantial contribution to the conception of the work and drafting of the work. All authors approved the final version to be published and agree to be accountable for all aspects of the work in ensuring that questions related to the accuracy or integrity of any part of the work are appropriately investigated and resolved.

Funding This work was supported by the Economic and Social Research Council (ESRC) Impact Acceleration Account (IAA).

Competing interests None declared.

Patient consent for publication Not required.

Ethics approval Ethical approval for the study has been provided by the School of Healthcare Sciences Research Governance and Ethics Committee, Cardiff University, UK.

Provenance and peer review Not commissioned; externally peer reviewed.

Data sharing statement No additional data available; that is, the dataset supporting the conclusions of this article is included within the article.
